# The nickel-chelator dimethylglyoxime inhibits human amyloid beta peptide in vitro aggregation

**DOI:** 10.1038/s41598-021-86060-1

**Published:** 2021-03-23

**Authors:** Stéphane L. Benoit, Robert J. Maier

**Affiliations:** 1 Department of Microbiology, The University of Georgia, 805 Biological Sciences Bldg, Athens, GA 30602 USA; 2 Center for Metalloenzyme Studies, The University of Georgia, Athens, GA 30602 USA

**Keywords:** Biochemistry, Drug discovery, Alzheimer's disease

## Abstract

One of the hallmarks of the most common neurodegenerative disease, Alzheimer’s disease (AD), is the extracellular deposition and aggregation of Amyloid Beta (Aβ)-peptides in the brain. Previous studies have shown that select metal ions, most specifically copper (Cu) and zinc (Zn) ions, have a synergistic effect on the aggregation of Aβ-peptides. In the present study, inductively coupled plasma mass spectrometry (ICP-MS) was used to determine the metal content of a commercial recombinant human Aβ_40_ peptide. Cu and Zn were among the metals detected; unexpectedly, nickel (Ni) was one of the most abundant elements. Using a fluorescence-based assay, we found that Aβ_40_ peptide in vitro aggregation was enhanced by addition of Zn^2+^ and Ni^2+^, and Ni^2+^-induced aggregation was facilitated by acidic conditions. Nickel binding to Aβ_40_ peptide was confirmed by isothermal titration calorimetry. Addition of the Ni-specific chelator dimethylglyoxime (DMG) inhibited Aβ_40_ aggregation in absence of added metal, as well as in presence of Cu^2+^ and Ni^2+^, but not in presence of Zn^2+^. Finally, mass spectrometry analysis revealed that DMG can coordinate Cu or Ni, but not Fe, Se or Zn. Taken together, our results indicate that Ni^2+^ ions enhance, whereas nickel chelation inhibits, Aβ peptide in vitro aggregation. Hence, DMG-mediated Ni-chelation constitutes a promising approach towards inhibiting or slowing down Aβ_40_ aggregation.

## Introduction

Alzheimer’s disease (AD), discovered more than a century ago by Lois Alzheimer^1^, is the most common cause of dementia in many elderly people, as well as in individuals with Down syndrome who survive beyond age 50. AD is a major health problem, in the United States and the rest of the world. According to the most recent national vital statistics report available in the USA (year 2017), AD is estimated to be the fifth cause of death for people aged 65 and over, and the third cause of death for people aged 85 and over, behind heart disease and cancer^2^. In the absence of a cure, and because of the overall aging population, a study from the Alzheimer’s Association predicts that by mid-century 13.8 million Americans will live with the disease, with one new case of AD developing every 33 s, resulting in nearly 1 million new cases per year. Based on the time of onset, AD is classified into two types: early-onset AD (EOAD), which typically develops before the age of 65, and late-onset AD (LOAD) for those older than 65^3^. In addition to intraneuronal tangles of hyperphosphorylated tau (τ) protein^4^, one hallmark of AD is characterized by various pathological markers in the brain, including accumulation of Amyloid Beta (Aβ) protein (in the form of senile plaques), as first proposed by Hardy and Higgins in a landmark study known as “the amyloid beta cascade hypothesis”^5^. Sequential proteolysis of the amyloid precursor protein (APP), an ancient and highly conserved protein^6^, by β-secretase and γ-secretase enzymes yields Aβ peptides of various lengths (38, 40 or 42 amino acids), depending upon the exact site of cleavage by the γ-secretase^7^. While the most abundant Aβ peptide is Aβ_40_, the most toxic is Aβ_42_^8^. The release of Aβ peptides is a normal physiological process. Indeed, not only Aβ peptides are naturally present in both the brain and the cerebrospinal fluid throughout the life of an individual^9–11^, they are also produced by cultured cells during normal metabolism^12^. However, once Aβ peptides form filamentous aggregates (e.g., amyloids), not only can they propagate their abnormal structures to the same precursor molecules (seeding), they can also propagate to other protein monomers (cross-seeding), such as that involved in Parkinson’s or Type 2 diabetes diseases^13^.

Alzheimer’s disease occurs sporadically in most cases; however, a sizable number of cases can be linked to mutations in various genes. For instance, mutations in the *APP* gene, or in genes encoding for enzymes involved in the APP processing (e.g. *PSEN1* or *PSEN2*), are predominantly associated with EOAD, whereas mutations in genes encoding for enzymes related to Aβ turnover, such as the apolipoprotein E (e.g. *APOE*), are usually associated with LOAD^14,15^. Besides genetic factors, environmental factors have been shown to play a role in AD, as revealed by a study on twins^16^. Environmental factors include toxic gases, such as CO, CO_2_, SO_2_ and NO_2_^17^, or metals, several of which have been shown to play a role on A*β* aggregation, fibrillization and toxicity, with potential implications on the progression of AD (for a recent review, see Liu et al.)^18^. The list includes heavy metals, such as aluminum (Al)^19^, cadmium (Cd)^20^ and mercury (Hg)^21,22^, and essential metals, such as copper (Cu) and zinc (Zn), and, to a lesser extent, iron (Fe)^23^. The role of Cu(I), Cu(II), or Zn(II) has been well documented^24–26^. Firstly, both A*β*_40_ and A*β*_42_ peptides have been shown to bind Cu(II) or Zn(II) with significant affinity in vitro, leading to A*β* aggregation^19,27–30^; secondly, a similar effect was observed in vivo, leading to plaque build-up and toxicity in AD animal models, for instance with Cu(II) in rabbits^31^, or with Zn(II) in mice^32^; thirdly, post-mortem analysis revealed that respective Cu, Fe and Zn levels in plaques of AD brains were 5.7, 2.8, and 3.1-fold higher compared to normal brains^33^; fourthly, accumulation of Cu and Zn co-localized with A*β* peptide deposits^34^. Taken together, these results have given birth to a theory known as the “metal hypothesis of AD”, that links metal homeostasis (especially that of Cu, Fe and Zn) and AD^35^. Recent discoveries on A*β* peptides-lipid interactions have confirmed the importance of metals in the onset and progression of AD: A*β* peptides can associate with cellular membranes, and A*β*-bound metals (especially Zn and Al) can blockade and disrupt Ca^2+^ channels, leading to neurotoxicity.

The logical follow up to these observations was the use of chelators to inhibit A*β* peptide aggregation, with the long-term goal of using metal chelation as therapeutic strategy for AD^36^. This research avenue has been investigated by several groups, with mixed outcomes. Chelators, such as EGTA, “tpen” (*N*,*N*,*N*′,*N*′-tetrakis(2-pyridyl-methyl) ethylene diamine), and bathocuproine have been shown to solubilize A*β* plaques from post-mortem brain tissue^37^. The 8-hydroxyquinoline derivatives Clioquinol and BPT-2, two copper-zinc chelators, have shown promising results in vitro^35,38^. Being able to cross the blood–brain-barrier (BBB), both have been tested in clinical trials, unfortunately the results appear inconclusive^39^. In another unrelated clinical trial, the rate of decline of daily living skills was significantly reduced in AD patients given desferrioxamine intramuscular twice daily for two years^40^. The authors originally attributed this effect to aluminum chelation, however desferrioxamine binds preferentially to iron (also copper and zinc, albeit with lower affinity); hence it is hard to draw firm conclusions about this trial. Alternative ways to target and modulate the toxicity of metal-bound (or metal-free) A*β* species include the use of (i) glycosylated polyphenols and their esterified derivatives, which present the advantage of using natural low toxicity compounds^41^; (ii) synthetic flavonoids and amino-isoflavones, which have shown promising results towards targeting metal sites^42^; (iii) small molecules, such as *N*^1^,*N*^1^‐dimethyl‐*N*^4^‐(pyridin‐2‐ylmethyl)benzene‐1,4‐diamine (“L2-b”) and its derivatives^43,44^; (iv) β-sheet breakers, which are small peptides (five amino-acids long) effective in reducing the Aβ_1-40_ aggregation, even in the presence of metal ions^45^.

In contrast to Cu, Fe and Zn, which are required cofactors for hundreds of enzymes, and fairly abundant in animals and humans^46^, nickel (Ni) does not appear to be needed in mammals, as mammalian hosts do not contain known Ni-dependent enzymes^47^. Furthermore, Ni levels are low, with less than 5 ppm (μg/g of ash) in most human organs, corresponding to less than 1% of the amount of Zn measured in the brain, heart, lung, or muscle, and less than 0.1% of the amount of Zn in the liver and kidney^48^. Even though Ni is rarely mentioned in association with A*β* peptides, a potential role for this transition metal should not be discarded. For instance, levels of A*β*_40_ and A*β*_42_ peptides were significantly increased (72–129%) in brains of mice exposed to a Ni-nanoparticle model of air pollution^49^, suggesting Ni might play a role in β-amyloid aggregation, at least in mice^49^. This finding prompted us to investigate the role of Ni in A*β* peptide aggregation, as well as the potential benefit of using the Ni chelator dimethylglyoxime (DMG) to slow down or even inhibit aggregation.

## Results

### Commercial recombinant A*β*_***40***_ peptide contains metals, including copper, zinc, and nickel

Most A*β* peptide preparations used for in vitro aggregation studies are synthetic (e.g. chemically synthesized) or recombinant peptides (*e.g.* expressed in prokaryotic or eukaryotic organisms). To determine what type of metals is associated with commercial human recombinant A*β* peptide, a A*β*_40_ peptide preparation was subjected to a twenty-element ICP-MS analysis (Table 1). Aluminum, copper, manganese and zinc were among the metals found in the A*β*_40_ peptide preparation, whereas iron was not detected. Surprisingly, the most abundant element associated with the recombinant A*β*_40_ peptide was selenium, followed by nickel (1 mg of Ni per g of A*β*_40_ peptide, corresponding to 0.073 moles of Ni per mole of peptide). Taken together, these results suggest that the recombinant A*β*_40_ peptide used in this study is already metal-bound upon commercialization; metals may be acquired during bacterial expression in the host (*E. coli*), during the purification process, or both. To our knowledge, it is the first time nickel is found in a commercial recombinant A*β*_40_ peptide purified preparation, suggesting the peptide (or aggregated peptides) can naturally coordinate the transition metal nickel, in addition to other metals such as aluminum, copper, manganese, selenium and zinc. The metal content of all the other kit components, including TBS, thioflavin and NaOH (used to resuspend the peptide) was also analyzed by ICP-MS (Table S1). Aluminum, manganese, iron, nickel copper, and zinc were detected in these components, however no selenium was detected. Besides, the bulk of nickel in the assay (reaction mix) was brought by the peptide (> 22-fold more Ni in A*β*_40_ compared to other kit components, see Table S1).

**Table 1:** ICP-MS metal analysis of commercial recombinant A*β*_40_-peptide.

Element	Metal/A*β*40 ratio	
	μg metal per g of A*β*_40_ peptide^a^	mmole metal per mole of A*β*_40_ peptide^b^
Lithium (^7^Li)	ND	ND
Beryllium (^9^Be)	ND	ND
Aluminum (^27^Al)	65.7	10.5
Vanadium (^51^V)	1.03	0.09
Chromium (^52^Cr)	15	1.25
Manganese (^55^Mn)	1.06	0.08
Iron (^56^Fe)	ND	ND
Cobalt (^59^Co)	ND	ND
Nickel (^60^Ni)	1,005	72.5
Copper (^65^Cu)	22.8	1.55
Zinc (^66^Zn)	45.7	3.07
Arsenic (^75^As)	ND	ND
Selenium (^82^Se)	27,784	1470
Rubidium (^85^Rb)	ND	ND
Strontium (^88^Sr)	3.6	0.18
Cadmium (^111^Cd)	ND	ND
Cesium (^133^Cs)	ND	ND
Barium (^137^Ba)	32.5	1.03
Lead (^207^Pb)	0.25	0.005
Uranium (^238^U)	ND	ND

*ND* not detected (below detection limit).

^a^background (water) subtracted-values.

^b^calculated with theoretical molecular mass of 4330 Da.

### Addition of nickel enhances A*β*_40_ peptide aggregation

**Figure 1:**
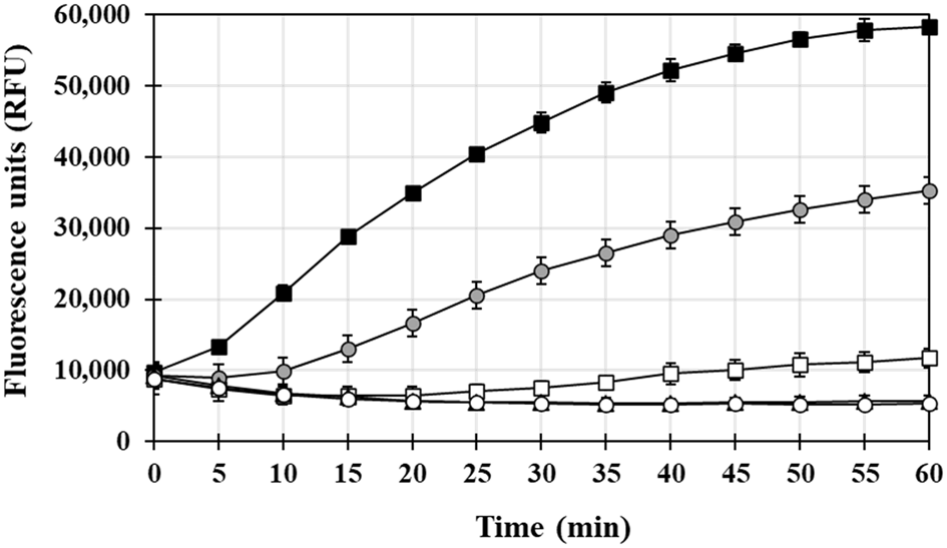
Time-dependent A*β*_40_ peptide aggregation in absence or presence of Ni(II) and DMG. A*β*_40_ peptide (40 µM) and thioflavin (40 µM) were mixed: in the absence of Ni(II) and DMG (white squares); with 100 µM Ni(II) (black squares); with 100 µM Ni(II) and 100 µM DMG (grey circles); with 100 µM Ni(II) and 500 µM DMG (black triangles); with 100 µM Ni(II) and 1 mM DMG (white circles). ThT-based fluorescence was measured every 5 min for 60 min. A ThT-only background control (no A*β*_40_ peptide) was included in the assay (data not shown). Results shown for each time point represent the mean and standard deviation (error bars) of background-subtracted values for triplicate wells. Results shown here correspond to results of experiment B, Table 2.

To determine the effect of nickel on A*β*_40_ peptide aggregation, a thioflavin-(ThT)-based aggregation kit was used in absence or presence of supplemental Ni(II) (Fig. 1 and Table 2). In absence of supplemental metal, a moderate but steady increase in A*β*_40_ peptide aggregation was observed (average fluorescence rate of 152 RFU/min, Fig. 1). We hypothesized this might be due to the intrinsic presence of metallic ions, including Cu^2+^, Ni^2+^ and Zn^2+^, as revealed by the ICP-MS metal analysis conducted in the present study (see above). Addition of 10 µM NiSO_4_ to the mixture increased the average aggregation rate by 2.5-fold (Table 1), while addition of 100 µM NiSO_4_ resulted in a dramatic 5.7-fold increase compared to the no supplemental metal control, suggesting the divalent cation Ni^2+^ can bind to the A*β*_40_ peptide and enhance its aggregation (Fig. 1 and Table 2). A similar effect was observed when NiCl_2_ was used (instead of NiSO_4_) as source of Ni^2+^ (data not shown); hence, the nature of the counterion does not appear to play a role in (or interfere with) the observed aggregation. Upon addition of 10 and 100 µM Zn(II), a fivefold and 14-fold increase in A*β*_40_ peptide aggregation rate was observed compared to the control, respectively (Table 2), in agreement with previously published studies^27–29^; in contrast, addition of 10 or 100 µM CuSO_4_ had no significant effect on the aggregation rate (Table 2). This result (lack of aggregation) could be due to the pH used in our study (pH 7.4). Indeed, Cu has been shown to induce A*β*_40_ peptide aggregation at acidic pH^30^, while at neutral pH it is known to promote mostly soluble dimers^50^.

**Table 2:** Recombinant human A*β*_40_ aggregation rate as a function of DMG and/or metal.

Supplemental metal (µM)	Supplemental DMG (µM)			
	0	100	500	1000
**Relative Aβ40 aggregation rate (% control)**				
A				
None (0)	**100**	33 ± 2	ND	ND
Ni (10)	252 ± 8	58 ± 11	ND	ND
Zn (10)	505 ± 29	457 ± 39	ND	ND
Cu (10)	103 ± 19	54 ± 12	ND	ND
B				
None (0)	**100**	15 ± 14	< 1	< 1
Ni (100)	567 ± 61	410 ± 91	< 1	< 1
Zn (100)	1423 ± 258	1468 ± 56	833 ± 156	328 ± 63
C				
None (0)	**100**	48 ± 8	15 ± 5	21 ± 10
Cu (100)	77 ± 2	66 ± 15	49 ± 8	40 ± 3

Three independent human A*β*_40_ peptide aggregation assays (A, B, C) were performed, with 25 µM A*β*_40_ peptide and 20 µM thioflavin (ThT) (A), or 40 µM A*β*_40_ and 40 µM ThT (B and C), in absence or presence of DMG (100, 500 or 1000 µM) and Ni, Zn, Cu (10 or 100 µM). ThT-based fluorescence (RFU) was measured every 5 min for 60 min and background fluorescence (ThT alone) was subtracted from all reactions. All reactions were done in triplicate. Results (relative A*β*_40_ peptide aggregation rate) represent the mean and standard deviation (n = 3) of the ratio (%) of the maximal aggregation rate (RFU per min) obtained for each indicated (metal, DMG) condition compared to the aggregation rate of the control (A*β*_40_ peptide only, no DMG, no metal, set as 100%, in bold). ND, not determined.

### Addition of DMG inhibits Aβ_***40***_ peptide aggregation

Addition of 100 µM of the Ni-specific chelator DMG in absence of supplemented metal severely reduced A*β*_40_ peptide aggregation, by 40 to 85% depending on experiments (Table 2). Furthermore, addition of 500 µM or 1000 µM DMG led to partial or full inhibition of the aggregation; in the latter case, we measured flat or even decreasing average fluorescence rates (reported as < 1% of control, Table 2). Hence this dose-dependent inhibitory effect suggests that (i) DMG is able to pull metals away from the A*β*_40_ peptide and (ii) the A*β*_40_ peptide aggregation observed in absence of supplemented metals is likely due to the intrinsic presence of metallic ions (including Ni^2+^) within the recombinant peptide preparation, since the addition of the chelator leads to inhibition. When increasing amounts of DMG were added to the reaction mixture in presence of 100 µM Ni^2+^, Cu^2+^ or Zn^2+^, results with mixed outcomes were obtained. Complete inhibition was observed in presence of Ni (Fig. 1 and Table 2) and only partial inhibition was seen in presence of Cu or Zn, however Zn was still able to induce A*β*_40_ peptide aggregation (Table 2). The respective efficacy (or lack thereof) of DMG in presence of Cu, Ni and Zn correlates with the chelator’s respective affinity for each metal, as revealed by mass spectrometry analysis of metal-DMG complexes (see below).

### Effect of pH on Aβ_***40***_ peptide aggregation in presence of metals or DMG

**Figure 2:**
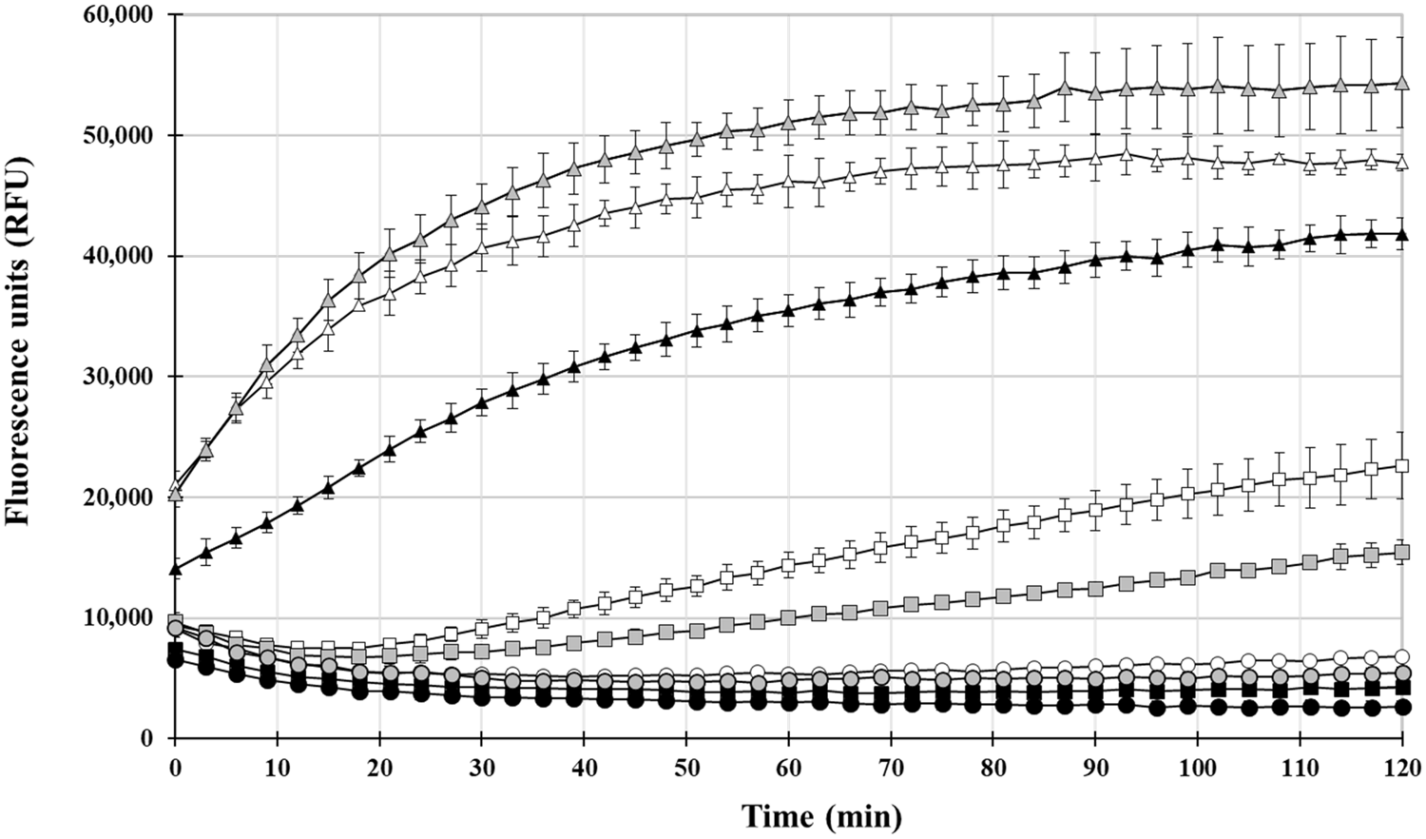
Time-dependent A*β*_40_ peptide aggregation in absence or presence of Ni(II) and DMG at various pHs. A*β*_40_ peptide (25 µM) and thioflavin (40 µM) were mixed in absence of metal or DMG (circles), in presence 25 µM Ni(II) (squares), or in presence of 10 µM Zn(II) (triangles). The final pH in the reaction was 6.5 (white symbols), 7.5 (grey symbols), or 8.5 (black symbols). For instance, black triangles represent RFUs measured in presence of Zn(II) at pH 8.5. ThT-based fluorescence was measured every 3 min for 120 min. A ThT-only background control (no A*β*_40_ peptide) was included in the assay (data not shown). Results shown for each time point represent the mean and standard deviation (error bars) of background-subtracted values for triplicate wells.

To study the effect of pH on A*β*_40_ peptide aggregation (in presence of metals or DMG), additional ThT-based aggregation experiments were conducted at pH 6.5, 7.5 or 8.5, with 25 µM A*β*_40_, in absence or presence of NiSO_4_ (25 µM), CuSO_4_ (25 µM), ZnSO_4_ (10 µM), or DMG (100 µM) (Fig. 2 and data not shown). Overall, aggregation rates at alkaline pH 8.5 (black symbols) were lower compared to pH 7.5 (grey symbols) or pH 6.5 (white symbols). As previously observed, addition of Zn(II) led to the fastest and sharpest increase in fluorescence (triangles) under all pHs tested. Interestingly, Ni(II)-induced aggregation (squares) was faster at pH 6.5, compared to pH 7.5, while it was absent at pH 8.5 (black squares). A*β*_40_ peptide aggregation in presence of Cu(II) or DMG was negligible under all 3 pH conditions tested (data not shown).

### Nickel binding to human recombinant Aβ_***40***_ peptide is confirmed by isothermal titration calorimetry

**Figure 3:**
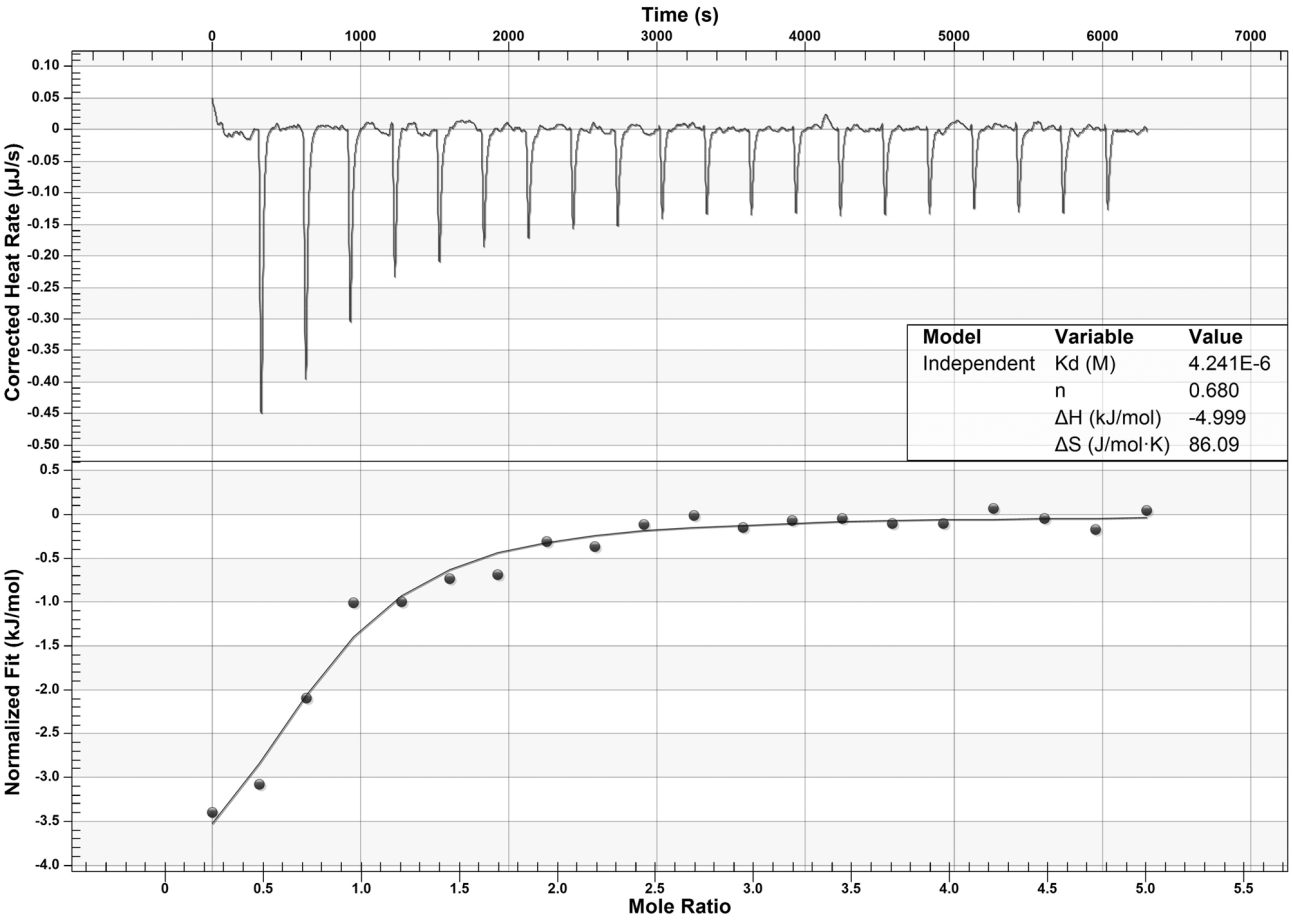
Isothermal titration calorimetry analysis of Ni binding to A*β*_40_. Top panel shows the raw data of heat release per injection, for 20 consecutive injections (2.38 µL) of NiSO_4_ (1 mM) into a 500-µL cell containing A*β*_40_ (20 µM). Bottom panel shows binding isotherms, obtained by integrating the areas of each injection peak. Data acquired with a NanoITC were analyzed using NanoAnalyze 1.2 software(TA Instruments). Shown in the inset are the best-fit values for the dissociation constant (*K*_*d*_), stoichiometry (n), enthalpic change (ΔH), and entropic change (ΔS).

Isothermal titration calorimetry (ITC) has been already used to analyze copper or zinc binding to various A*β* peptides, including A*β*_40_^51–53^. In the current study, we used ITC to determine whether nickel can bind to the A*β*_40_ peptide. The peptide (same used in ThT-based aggregation assays) was present in the sample cell at a concentration of 20 μM. Twenty injections of NiSO_4_ (1 mM solution, 5 μM increments in sample cell) were performed every 5 min under constant stirring (350 rpm) at 25 °C, and the heat release was measured (Fig. 3). The heat release profile indicates Ni binding to the peptide (Fig. 3, top Panel). The best fit of Ni titration (Fig. 3, bottom Panel) suggests an apparent stoichiometry of less than 1 mol Ni(II) per mole of A*β*_40_ peptide (~ 0.7), in range with previously reported stoichiometry ratios of 1:1 for Cu(II) or Zn(II), and A*β*_40_^54^. The apparent K_d_ value for Ni is approximately 4.2 μM, similar to that previously reported of 7 ± 3 μM for Zn^53^. Furthermore, the ΔH (enthalpy) and ΔS (entropy) were found to be  − 5 kJ/mol and 86 mol/J/K, suggesting the Ni-A*β*_40_ binding event can be considered both exothermic and spontaneous. Injection of DMG (instead of nickel) into the sample cell containing A*β*_40_ peptide did not induce any significant change, indicating that DMG cannot bind to the peptide (data not shown). Hence, this result suggests the inhibitory effect of DMG on A*β*_40_ peptide aggregation, as observed with ThT-based assays, is due to nickel chelation, rather than direct DMG-A*β*_40_ peptide inhibitory interaction.

### DMG-metal complexes can be detected by FTICR-MS

Aqueous solutions containing only DMG, or DMG in combination with Ni, Cu, Fe, Se or Zn salts were analyzed using Fourier Transform Ion Cyclotron Resonance Mass Spectrometry (FTICR-MS). In absence of added metal, two monomeric isoforms were detected, corresponding to either DMG, H^+^ (117.06585 m/z) or DMG, Na^+^ (139.04780 m/z) (Supplementary Fig. S1). In presence of Ni, complexes consisting of two DMG and one Ni, with either H^+^ (289.04412 m/z) or Na^+^ (311.02607 m/z) were detected. This was expected, as two DMG are required to chelate one Ni (Supplementary Fig. S1). Surprisingly, FTICR-MS analysis of a DMG-Ni aqueous solution further revealed the presence of [DMG]_4_-(Ni)_2_ complexes, mostly in the Na + form (599.06292 m/z) (Supplementary Fig. S1). While [DMG]_2_-Cu complexes were identified, only monomeric DMG (H^+^ or Na^+^) was observed in presence of Fe, Zn or Se; no dimeric or tetrameric DMG-Se or DMG-Zn complexes could be detected, suggesting DMG does not coordinate with Fe, Zn or Se (Supplementary Fig. S1).

### Analysis of DMG in brain samples using FTICR-MS and NMR

The fact that DMG inhibits A*β*_40_ peptide aggregation in vitro suggests it might be able to do the same in vivo, however DMG would first need to cross the blood-brain barrier (BBB). To determine whether DMG can localize to the mouse brain, we used FTICR-MS (see above) and Nuclear Magnetic Resonance (NMR). NMR was successfully used to detect DMG in the livers of mice subjected to daily oral doses (6. 1 mg) of aqueous DMG for 3 days^55^. In the present study, the same treatment was administered (*e.g.* one daily oral delivery for 3 days), brain samples were processed and analyzed by NMR and FTICR-MS and compared to brain samples from (no DMG) control mice. Unfortunately, both methods failed to identify DMG (whether by itself or metal-chelated) in brain samples.

## Discussion

To study A*β* peptide in vitro aggregation, one can choose to use either chemically synthetic peptides, or recombinant peptides, expressed in, and purified from, organisms such as *E. coli*. Synthetic A*β* preparations have been associated with various problems, such as presence of impurities in the preparation, incorporation of the L-form of amino-acids (*e.g.* D-His, D-Met, D-Arg) instead of the L-form during synthesis, or reproducibility issues in terms of quality and yield, to a point that even batch-to-batch variations have been reported^56–58^. On the other hand, the expression and purification of recombinant A*β* peptides also bring their own limitations and issues, including low yield, reduced solubility and presence of oxidized amino-acids (e.g. Met_35_-sulfoxide)^58^. One major difference between synthetic and recombinant A*β* peptides though, often overlooked in the literature, is the absence and presence of metals associated with each preparation, respectively. Indeed, any protein or peptide showing natural affinity for one (or several) metal(s), as it is the case with A*β* peptides for copper or zinc^24^, will likely encounter (and bind to) the metals within the host (*E. coli* or other hosts). Hence recombinant A*β* peptides are likely to be already associated with metals upon purification, in contrast to synthetic peptides. Given that both Cu and Zn enhance A*β* peptide aggregation, one can expect that Cu or/and Zn-containing recombinant A*β* peptide will be “naturally” more prone to aggregation than their synthetic counterparts. This could account for differences reported in a study by Finder and coworkers, who found that recombinant A*β*_42_ peptides (likely metal-bound) aggregated faster and were more neurotoxic than synthetic A*β*_42_ peptides (likely metal-depleted)^58^. In order to validate our hypothesis (*e.g.* recombinant A*β* peptides are metal-rich) we subjected a commercial recombinant A*β*_40_ peptide preparation to ICP-MS metal analysis. Results unambiguously showed the presence of various metals, including Al, Cu, Mn, Zn, Se and Ni, the two latter elements being by far the most abundant (ppm range). Additional metal analysis of other components of the commercial kit revealed the presence of Al, Cu, Fe, Mn, and Ni, but no Se; furthermore, components-associated Ni levels were negligible compared to the peptide-associated Ni levels. Metallic ions, more especially Cu(II) and Zn(II), have been shown to enhance in vitro aggregation of both A*β*_40_ and A*β*_42_ peptides^19,27–30^. Ni(II) can now be added to the list of A*β* peptide-aggregating metals, based on results from the present study. Indeed, our thioflavin-based assays revealed that Ni(II) enhance A*β*_40_ aggregation, whereas DMG-mediated Ni-chelation inhibits it . Moreover, Ni(II) was found to be more efficient than Cu(II), and less efficient than Zn(II), respectively, at promoting A*β*_40_ aggregation, under the conditions tested in our study. Since various parameters (such as pH and temperature) have been previously shown to have an effect on metal-induced aggregation^30,59^, we tested the effect of pH on Ni-dependent aggregation. Three buffers with similar salt content (192 mM NaCl) but various pHs (6.5, 7.5, or 8.5) were used. Interestingly, acidic pH (6.5) conditions increased Ni-induced aggregation compared to the control pH (7.5), whereas Ni-induced aggregation was abolished at pH 8.5. The increased Ni-induced aggregation at acidic pH, as observed in the present study, is in agreement with previous published data from Atwood et al., who reported an increase of A*β*_1–40_ aggregation in presence of 1 μM Ni at pH 6.6, compared to pH 7.4^30^. Likewise, the same study correlated acidic pH (6.6) with enhanced aggregation, in presence of either Cu or Zn (both at 20 μM). Herein, Zn-induced aggregation was slightly higher at pH 7.5 compared to 6.5, and significantly faster compared to pH 8.5. The effect of pH on Cu-induced aggregation was negligible, but it is worth noting that the effect of Cu was very limited throughout our ThT-based assays, for a reason yet to be determined. The effect of temperature on Ni-dependent A*β*_40_ aggregation was not tested with the fluorescence-based method, as all assays were carried out at 37 °C. However, Ni-A*β*_40_ binding was also observed at 25 °C, as shown by ITC (see below). Although results obtained with both methods cannot be directly compared nevertheless we can report that Ni binding (to A*β*40) happens both at 25 °C and 37 °C.

Since aggregation in presence of a particular metal (*e.g.,* nickel) suggests initial metal-peptide binding, we further investigated the likelihood of Ni binding to A*β*_40_, by using ITC. The calorimetry-based method has been successfully used in the past to study Zn binding to A*β*_40_, both at low (10 μM) and high (70 μM) concentrations^53^. In the current study, we only looked at the effect of Ni on low A*β*_40_ concentration, with a starting concentration of A*β*_40_ in the sample cell at 20 μM. After Ni was injected via 20 consecutive injections, every 5 min (5 μM increments), a heat profile characteristic of independent metal-binding was observed. Although the apparent K_d_ (4.2 μM) is similar to that reported for Zn^53^, the apparent stoichiometry (0.7 mol of Ni per mole of A*β*_40_) is significantly lower than that previously reported by Drochioiu and colleagues, who found that synthetic A*β*_40_ peptide displays high affinity toward nickel ions with up to three Ni^2+^ ions bound per A*β*_40_ peptide^60^.However, the discrepancy between our results and theirs could be due to the nature of A*β*_40_ peptide used, and the type of analytical method used to analyze Ni-A*β*_40_. In our study, we used a purified recombinant A*β*_40_ peptide, and ITC, whereas Drochioiu et al. used synthetic A*β*_40_ peptide, electrospray ion trap mass spectrometry (ESI–MS) and circular dichroism (CD). Nevertheless, results from both groups indicate that A*β*_40_ can bind nickel with high affinity. Furthermore, our results confirm that DMG inhibits A*β*_40_ aggregation through Ni chelation (not direct contact with the peptide), since titration of the peptide with DMG did not induce any peptide conformational change, as observed with ITC.

Given the presence of Cu^2+^, Ni^2+^, Zn^2+^ in recombinant A*β*_40_ peptide, combined to their respective effect on A*β*_40_ peptide aggregation, metal chelation therapy towards AD constitutes a valid approach. However, the risk of chelation therapy is that removal of essential metal ions will lead to serious adverse effects (for instance, iron-deficiency anemia) as pointed out by other researchers^35^. Hence it is preferable to use chelators with select affinity towards non-essential metals: the Ni-specific chelator DMG is therefore a good candidate. Indeed, DMG has been used for many years to detect, quantitate or decrease Ni levels in various environments; it can also be used to inhibit the growth of bacteria, including multidrug resistant Enterobacteriaceae, as recently demonstrated by our group^55^. Mammalian hosts do not contain known Ni-dependent enzymes, which makes Ni-chelation therapy an attractive approach^47^. On the other hand, most bacteria, including pathogenic ones, require nickel as cofactor for one or several enzymes, such as [Ni–Fe] hydrogenase(s)^61^ or urease^47^. Thus, DMG-mediated inhibition of these enzymes, as demonstrated with *Salmonella* Typhimurium hydrogenases or *Klebsiella pneumoniae* urease, leads to bacterial growth inhibition, both in the mouse and in the wax moth animal models^55^. In the present study, we showed that DMG can drastically reduce, and even abolish A*β*_40_ peptide aggregation. The inhibitory effect was observed in absence of supplemental metal, as well as in presence of copper, nickel or even zinc (albeit with lower efficacy). Although thioflavin is a popular reporter of amyloid aggregation, it mostly binds to *β*-sheet rich fibrils^62,63^. Therefore, our conclusions on the effect of Ni and Ni-chelation (DMG) on A*β* peptide-aggregation must be limited at this time to the *β*-sheet content. Additional experiments will be needed to determine whether Ni and its chelator have a broader effect on other A*β* peptide conformations. Likewise, further experiments will be conducted to test whether DMG can inhibit the aggregation of other physiologically relevant A*β* peptides, for instance A*β*_42_.

The current study is not the first one to report inhibitory effect of a nickel chelator on A*β* peptide aggregation. Indeed, Reinhardt and colleagues reported beneficial effects of the nickel chelator disulfiram on AD hallmarks, including inhibitory effects on A*β*_42_ peptide aggregation^64^. The study however was not aimed at establishing any link between Ni and AD. The authors found that disulfiram increased synthesis of the metalloproteinase α-secretase, resulting in secretion of the neuroprotective APP cleavage product sAPPα and thus preventing formation of the amyloidogenic *β*A peptides^64^. The concentration of disulfiram shown to have inhibitory effects on peptide aggregation was significantly lower compared to DMG concentrations used in our study, however the disulfiram drug is highly toxic, even at low doses, with concentrations higher than 5 μM inducing cytotoxicity^64^. This finding correlates with previous studies linking disulfiram with negative outcomes, such as elevated nickel levels in rat brains^65^, elevated nickel levels in body fluids of patients with chronic alcoholism^65,66^, as well as hepatotoxicity in humans^67,68^.

If DMG were to be used in a clinical trial against AD, it might not only inhibit A*β* plaque formation, but also Ni-requiring microorganisms. This, however, would not necessarily be a negative outcome, in light of the link between pathogens and AD, known as the “infection hypothesis of AD”^69–71^. The list of pathogens potentially linked to AD includes viral, fungal and bacterial species. Among bacteria directly or indirectly associated with AD, one can find *Helicobacter pylori*^72,73^, *E. coli*^74^ and *Salmonella* Typhimurium^75^, all of which require Ni as cofactor for one or several enzymes (for a review, see Maier and Benoit^47^). In the case of *H. pylori*, another protein is relevant to the pathogen/AD link. The gastric pathogen produces abundant amounts (2% of total protein) of a small histidine-rich protein (Hpn) that has been shown to develop amyloid-like fibrils in vitro^76^. The continuous production of Hpn by the bacterium during decades of chronic gastric infection could result in leakage of the protein, first into the bloodstream and eventually into the brain, potentially triggering AD, as hypothesized by Ge and Sun^77^. More generally, an antimicrobial role for A*β* peptides (as part of the brain’s ancient immune system) has been proposed, as part of a “new amyloidogenesis model”^78^. The model is based on findings by Kumar and colleagues, who reported ^75^ that bacterial infection of the brains of transgenic mice result in accelerated A*β* plaque deposition, closely colocalizing with the invading bacteria (in this case, *Salmonella*)^75^.

**Figure 4:**
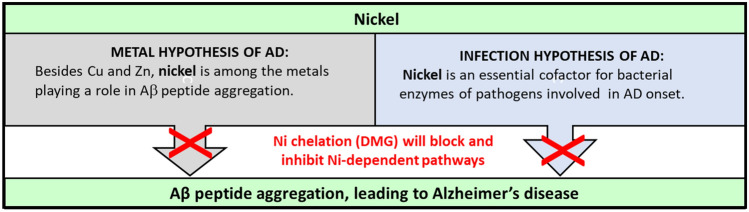
Hypothetical model showing a dual role for nickel (Ni) and a proposed mode of action for DMG-mediated Ni chelation. Ni can bind to A*β* peptides, leading to aggregation and plaque formation (left side, metal hypothesis of AD). In addition, Ni is required as cofactor for enzymes (such as hydrogenase and urease) of pathogens previously shown to play a role in A*β* peptide aggregation (right side, infection hypothesis of AD). The Ni-chelator DMG could inhibit A*β* peptide aggregation and the progression of AD (red crosses), either directly (left side) or indirectly (through pathogen inhibition, right side).

In summary, Ni could affect the onset and the progression of AD through two different mechanisms, as depicted in our proposed model (Fig. 4). The first mechanism involves the binding of Ni^2+^ to A*β* (A*β*_40_, possibly A*β*_42_), eventually leading to aggregation, plaque formation and AD; this would comply with the metal hypothesis of AD. The second mechanism involves the use of Ni^2+^ as a required cofactor for various enzymes (*e.g.* Ni-glyoxalase, Ni-superoxide dismutase, Ni-acireductone dioxygenase, [NiFe] hydrogenases and urease, see^47^) of pathogens previously shown to play a role in A*β* peptide aggregation; alternatively some of these pathogens might contribute to AD independently of A*β* plaque formation. Both scenarios would fit the “infectious hypothesis of AD”. Whether one Ni-dependent mechanism is preferred over the other, or both actively contribute to the onset and/or the progression of AD, nevertheless a DMG-mediated Ni-chelation strategy is at the intersection of both (the metal and the infectious) hypotheses. Thus, it is likely to interfere and disrupt both mechanisms, eventually slowing down or stopping the progression of AD. More than a century after Alois Alzheimer fist described the disease, and without any cure on the horizon, new therapeutic strategies are urgently needed to combat this neurodegenerative disease; we believe that nickel chelation (via DMG treatment) is a promising AD-combatting strategy that warrants further research.

## Materials and methods

### Chemicals

The water-soluble form (2Na, 8H_2_O) of DMG was used in this study (ref # 40400, Honeywell-Fluka, Muskegon, MI, USA). All metals (CuSO_4_, FeSO_4_, Na_2_SeO_3_, NiCl_2_, NiSO_4_, ZnSO_4_) are from Sigma-Aldrich (Saint Louis, MO, USA).

### Amyloid beta metal analysis

Metal levels for 20 elements (Li, Be, Al, V, Cr, Mn, Fe, Co, Ni, Cu, Zn, As, Se, Rb, Sr, Cd, Cs, Ba, Pb, U) were determined by inductively coupled plasma mass spectrometry (ICP-MS). Briefly, 0.5 mg of lyophilized human recombinant A*β*_40_ peptide (expressed in *E. coli*, purified and manufactured by rPeptides, Watkinsville, GA) was resuspended in ultrapure water to a final concentration of 5 mg/mL, digested overnight with concentrated trace metal grade nitric acid, heated for 2 h at 95 °C and subjected to ICP-MS using a Thermo X-Series II ICP-MS (Center for Applied Isotope Studies, University of Georgia, Athens, GA). The same treatment and analysis were performed on three other kit components: TBS 10X (reaction buffer), Thioflavin stock (400 μM) and NaOH 10 mM (used to resuspend the A*β*_40_ peptide).

### Amyloid beta aggregation

The effect of metals, DMG, and/or pH on human recombinant A*β*_40_ aggregation was monitored using a thioflavin T (ThT)-based kit, following the manufacturer’s recommendation (kit# A-1180-1, rPeptides, Watkinsville, GA, USA). This kit contains human recombinant A*β*_40_ peptide (> 97% pure, as determined by manufacturer’s HPLC) with the following sequence DAEFRHDSGYEVHHQKLVFFAEDVGSNKGAIIGLMVGGVV, as provided by the manufacturer. Briefly, standard assays were conducted in triplicate in black polystyrene 96-well plates, in presence of Tris Buffer Saline (TBS) pH 7.4, or TBS pH 8.5, or 2-(*N*-morpholino)ethanesulfonic acid (MES) buffer saline pH 6.5, Th-T (20 or 40 μM), and human recombinant A*β*_40_ peptide (25 or 40 μM), with or without DMG (100, 500, or 1000 μM), CuSO_4_, NiSO_4_ or ZnSO_4_ (10, 25, or 100 μM). The aggregation of A*β*_40_, as shown by the increase in fluorescence (λex = 440 nm/λem = 485 nm) over time, was followed for 60 min or 120 min, with reading every 3 or 5 min, using a Synergy MX reader (Biotek, Winooski, VT). A ThT-only background control (no A*β*_40_ peptide) was included in triplicate in all experiments and subtracted from all readings. The aggregation rate, defined as the increase in fluorescence per min (RFU/min), was calculated by using the formula [A(_440, 485)_ at T_40 min_) − A_(440, 485)_ at T_20 min_]/20. These time points (20 to 40 min) were chosen because they constantly displayed the best linearity in every assay. Results shown are expressed as (mean and standard deviation of) percentages; they represent the ratio of aggregation rate (RFU/min) for a given (DMG, metal) condition, compared to the aggregation rate obtained for the control (A*β*_40_ peptide, no DMG, no metal added, set as 100% for each experiment).

### Isothermal titration calorimetry

Binding assays of A*β*_40_ peptide and Ni or DMG were performed using a Nano ITC calorimeter (TA instruments, New Castle, DE). Briefly, 1 mg of lyophilized A*β*_40_ peptide (# A-1157-2, rPeptides) was resuspended with 1% NH_4_OH to a concentration of 250 μM, sonicated for 15–20 s, before being diluted to a final concentration of 20 μM A*β*_40_ using ddH_2_O and TBS 10X, pH 7.4 (working buffer: NH_4_OH 0.2%, TBS 1X, pH 7.4 (“NTBS”)). A volume of 500 μL was loaded onto the ITC sample cell, and the injection syringe was filled with 50 μL of either NTBS buffer (control), 1 mM NiSO_4_, or 1 mM DMG. All samples were degassed for 15 min at 25 °C before use. Titration was initiated using a program for 20 injections (2.38 μL each, every 5 min) with continuous stirring (350 rpm) at constant temperature (25 °C). ITC data were analyzed using NanoAnalyze 1.2 software (TA Instruments). Data obtained with the control experiment (A*β*_40_ peptide in sample cell, buffer in syringe) were subtracted from each experiment to account for any injection-related heat change. The Ni-A*β*_40_ experiment was done in triplicate, with a representative data set shown in figures.

### Analysis of DMG and DMG-metal complexes in commercial preparations

Aqueous solutions of DMG (0.5 mg/mL or 1.6 mM), with or without Cu, Fe, Ni, Se or Zn solutions (0.16 mM each) were analyzed by Fourier Transform Ion Cyclotron Resonance Mass Spectrometry (FTICR-MS), using a Bruker Solari X ESI/MALDI-12 T FT-ICR high precision mass spectrometer (Proteomics and Mass Spectrometry Facility, University of Georgia). The pH of all aqueous DMG solutions, with or without metal, was approximately 11. All samples were mixed (1:1) with methanol prior to injection.

### Detection of DMG and DMG-metal complexes in mouse brains

All procedures were performed in accordance with the relevant guidelines and regulations and approved by the University of Georgia IACU committee, and the study was carried out in compliance with the ARRIVE guidelines (http://www.nc3rs.org.uk/page.asp?id=1357). A group of 6 (C57/BL) mice was used for this experiment: 3 mice were given 0.2 mL of 100 mM DMG (~ 6.1 mg) every day for three days and 3 mice were used as (no DMG) controls. Mice were euthanized by CO_2_ asphyxiation and cervical dislocation. Brains were quickly removed and frozen at  − 80 °C. Upon thawing, brains were cut into pieces and homogenized in 2 mL sterile deionized water, incubated for 1 h at 90 °C, sonicated for 20 s and spun down (16,800 × g for 6 min). Supernatants were passaged through a 0.45 μm filter unit and analyzed by FTICR-MS (see above) and Nuclear Magnetic Resonance (NMR), as previously described ^55^.

## Supplementary Information

Supplementary Information 1.

Supplementary Information 2.
