# Supplementary material for: The nickel-chelator dimethylglyoxime inhibits human amyloid beta peptide in vitro aggregation
Source: Sci Rep. 2021 Mar 23;11:6622. doi: 10.1038/s41598-021-86060-1 (PMC7988135; doi:10.1038/s41598-021-86060-1)
Supplement: Supplementary file 1 — Supplementary Information 1. [file 41598_2021_86060_MOESM1_ESM.pptx]

## Slide 1
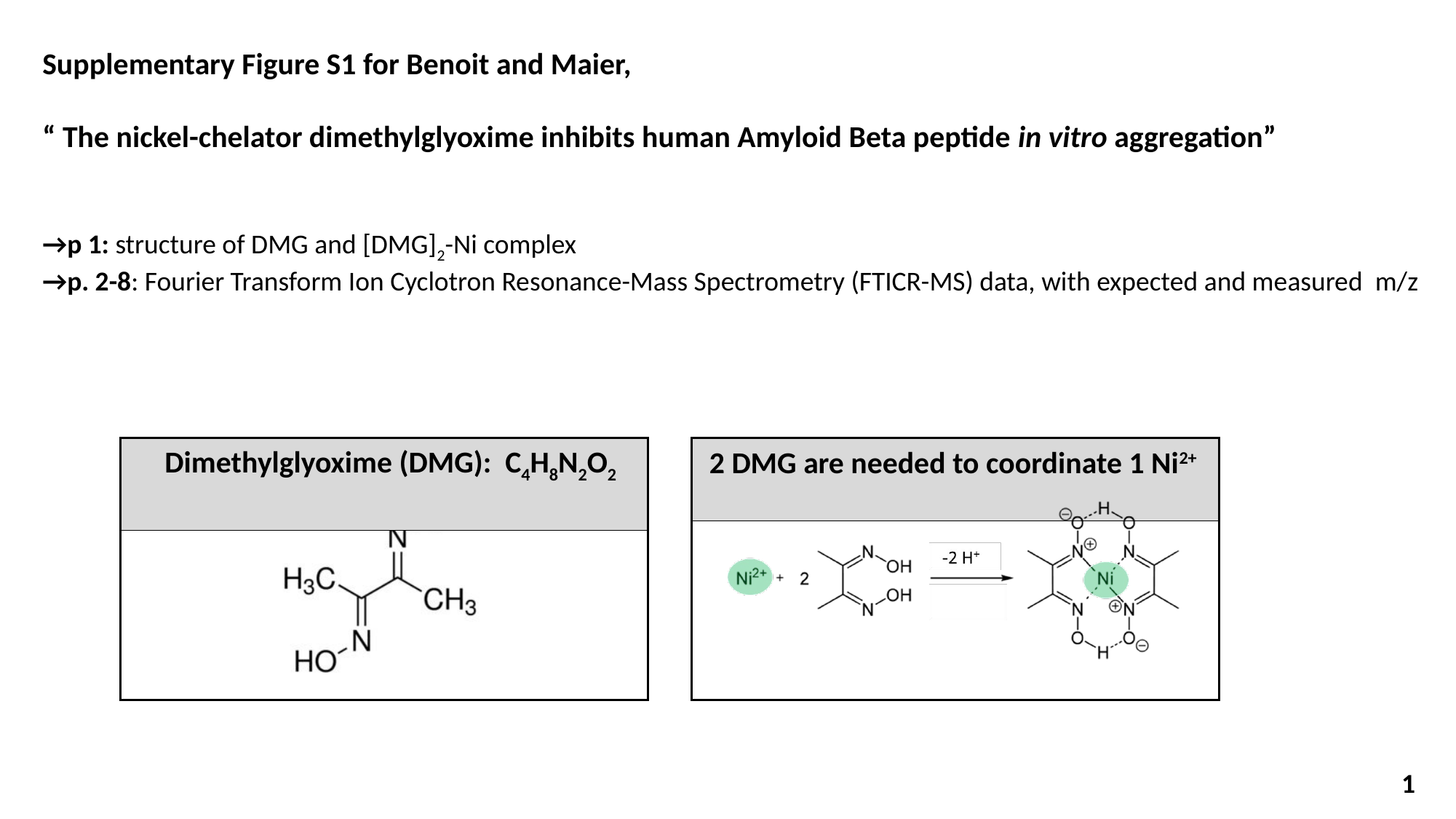

Supplementary Figure S1 for Benoit and Maier,
“ The nickel-chelator dimethylglyoxime inhibits human Amyloid Beta peptide in vitro aggregation”
→p 1: structure of DMG and [DMG]2-Ni complex
→p. 2-8: Fourier Transform Ion Cyclotron Resonance-Mass Spectrometry (FTICR-MS) data, with expected and measured m/z
 Dimethylglyoxime (DMG): C4H8N2O2
 2 DMG are needed to coordinate 1 Ni2+
1

## Slide 2
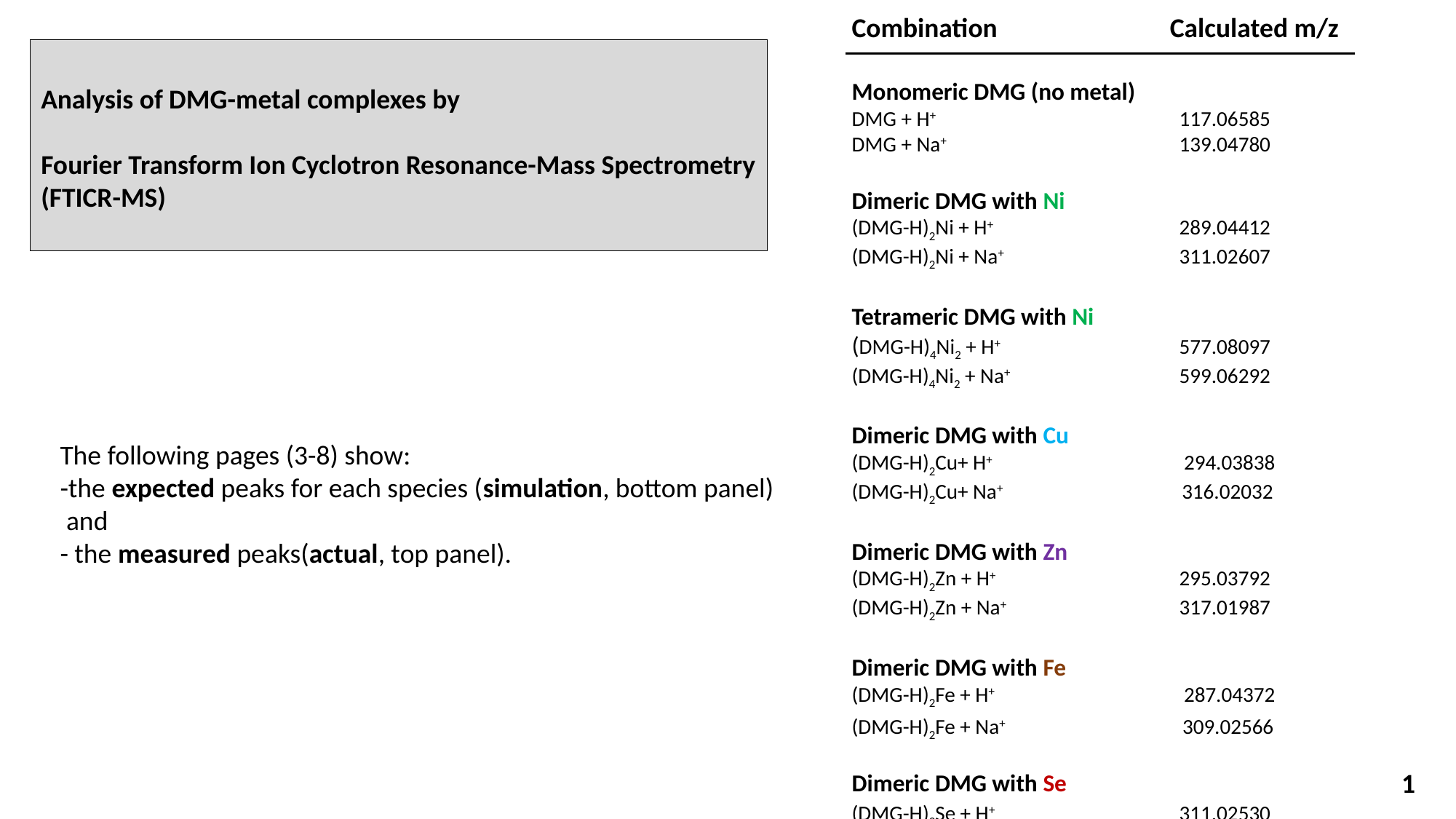

Combination Calculated m/z
Monomeric DMG (no metal)
DMG + H+			117.06585
DMG + Na+			139.04780
Dimeric DMG with Ni
(DMG-H)2Ni + H+		289.04412
(DMG-H)2Ni + Na+		311.02607
Tetrameric DMG with Ni
(DMG-H)4Ni2 + H+		577.08097
(DMG-H)4Ni2 + Na+		599.06292
Dimeric DMG with Cu
(DMG-H)2Cu+ H+		 294.03838
(DMG-H)2Cu+ Na+		 316.02032
Dimeric DMG with Zn
(DMG-H)2Zn + H+		295.03792
(DMG-H)2Zn + Na+		317.01987
Dimeric DMG with Fe
(DMG-H)2Fe + H+		 287.04372
(DMG-H)2Fe + Na+		 309.02566
Dimeric DMG with Se
(DMG-H)2Se + H+		311.02530
(DMG-H)2Se + Na+		333.00725
Analysis of DMG-metal complexes by
Fourier Transform Ion Cyclotron Resonance-Mass Spectrometry
(FTICR-MS)
The following pages (3-8) show:
-the expected peaks for each species (simulation, bottom panel)
 and
- the measured peaks(actual, top panel).
1

## Slide 3
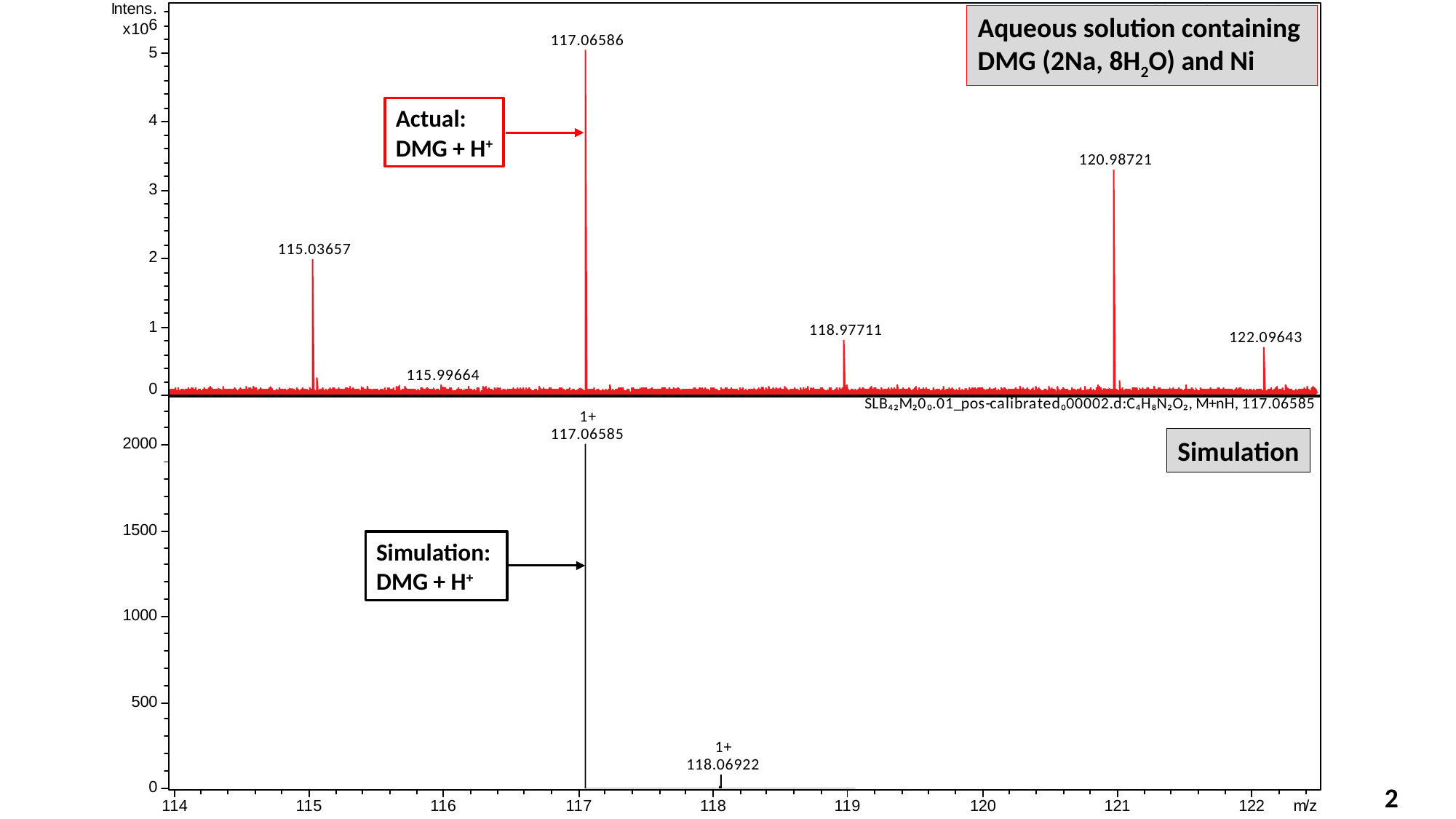

Aqueous solution containing
DMG (2Na, 8H2O) and Ni
Actual:
DMG + H+
Simulation
Simulation:
DMG + H+
2

## Slide 4
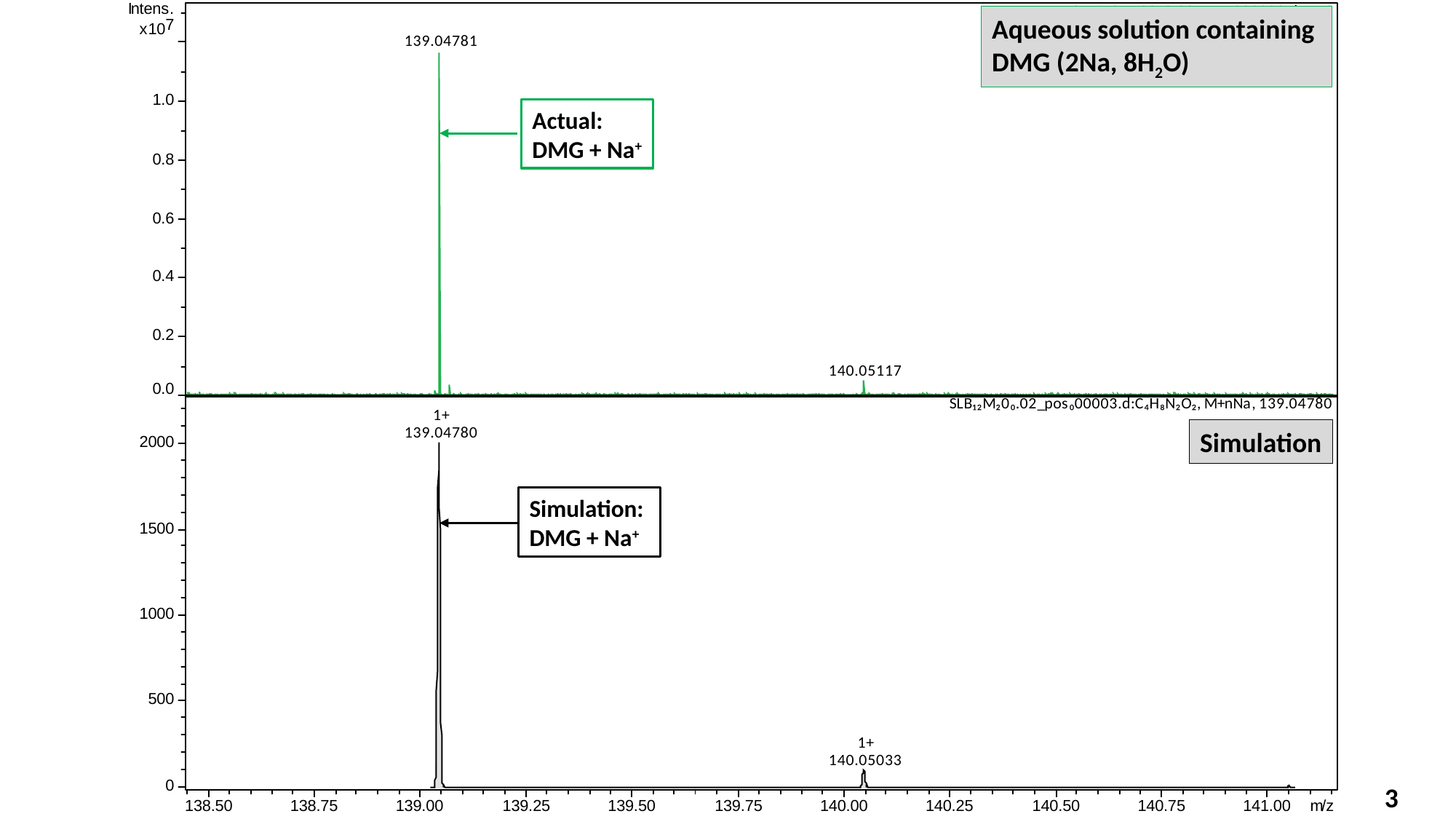

Aqueous solution containing
DMG (2Na, 8H2O)
Actual:
DMG + Na+
Simulation
Simulation:
DMG + Na+
3

## Slide 5
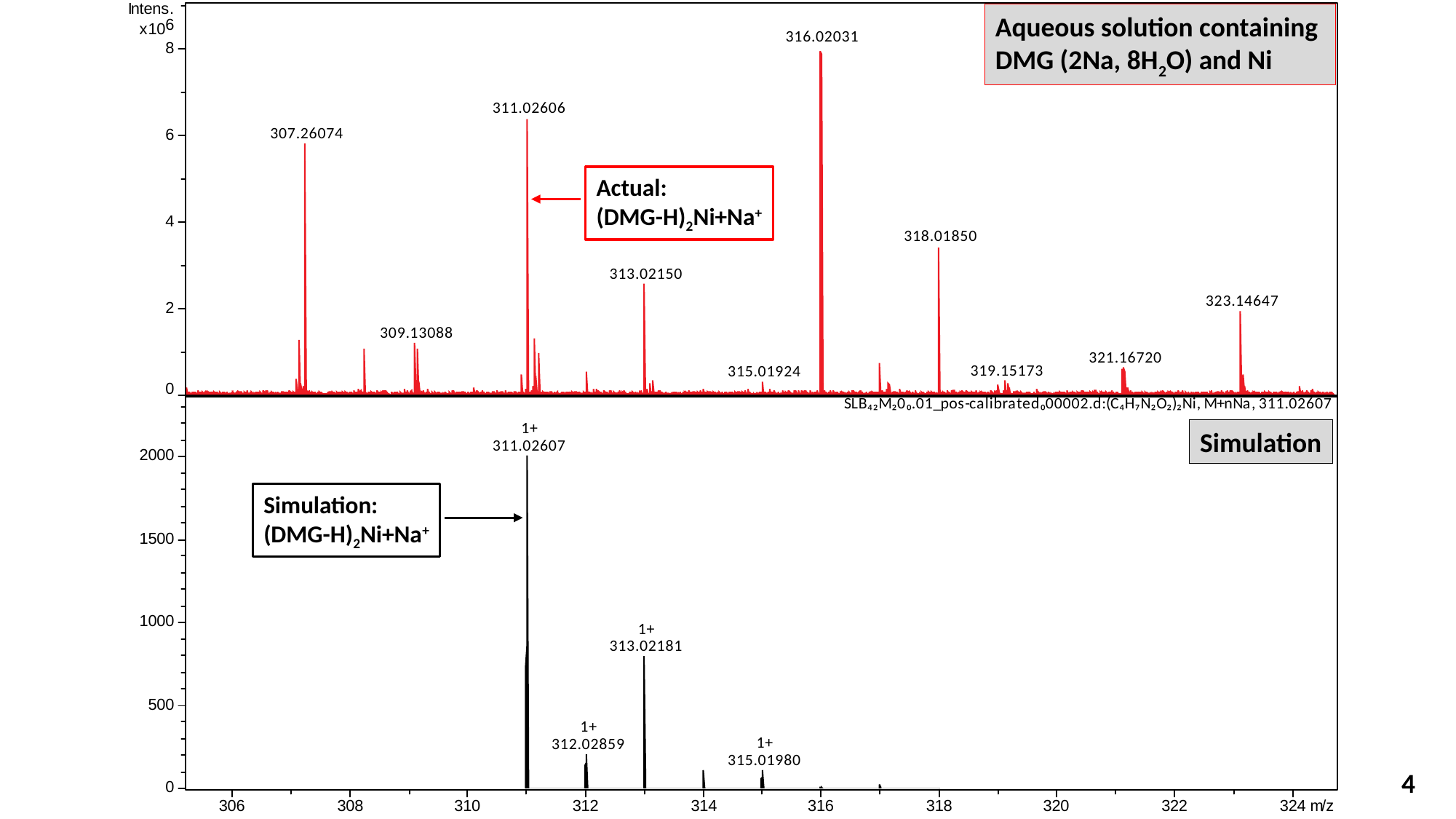

Aqueous solution containing
DMG (2Na, 8H2O) and Ni
Actual:
(DMG-H)2Ni+Na+
Simulation
Simulation:
(DMG-H)2Ni+Na+
4

## Slide 6
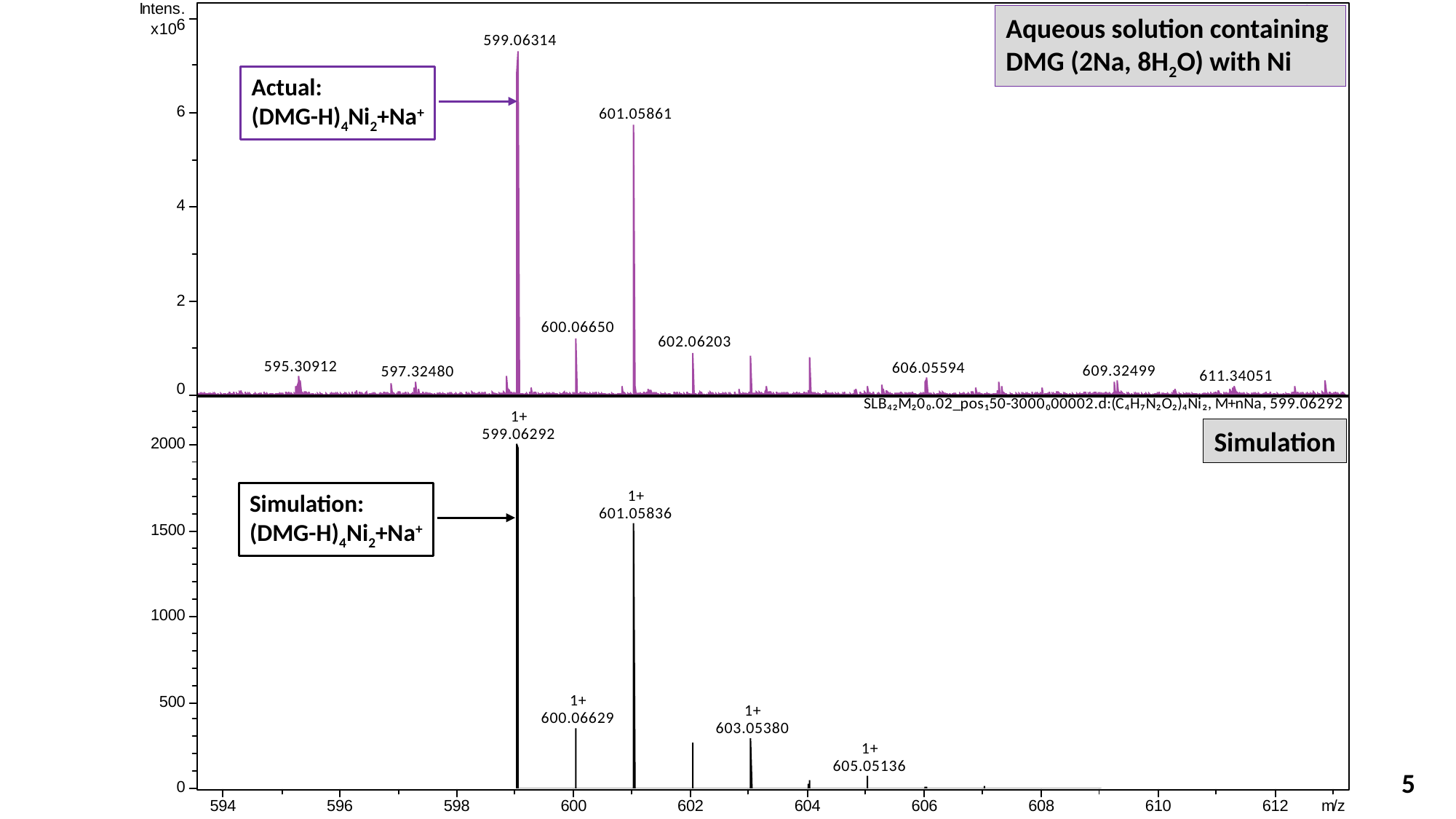

Aqueous solution containing
DMG (2Na, 8H2O) with Ni
Actual:
(DMG-H)4Ni2+Na+
Simulation
Simulation:
(DMG-H)4Ni2+Na+
5

## Slide 7
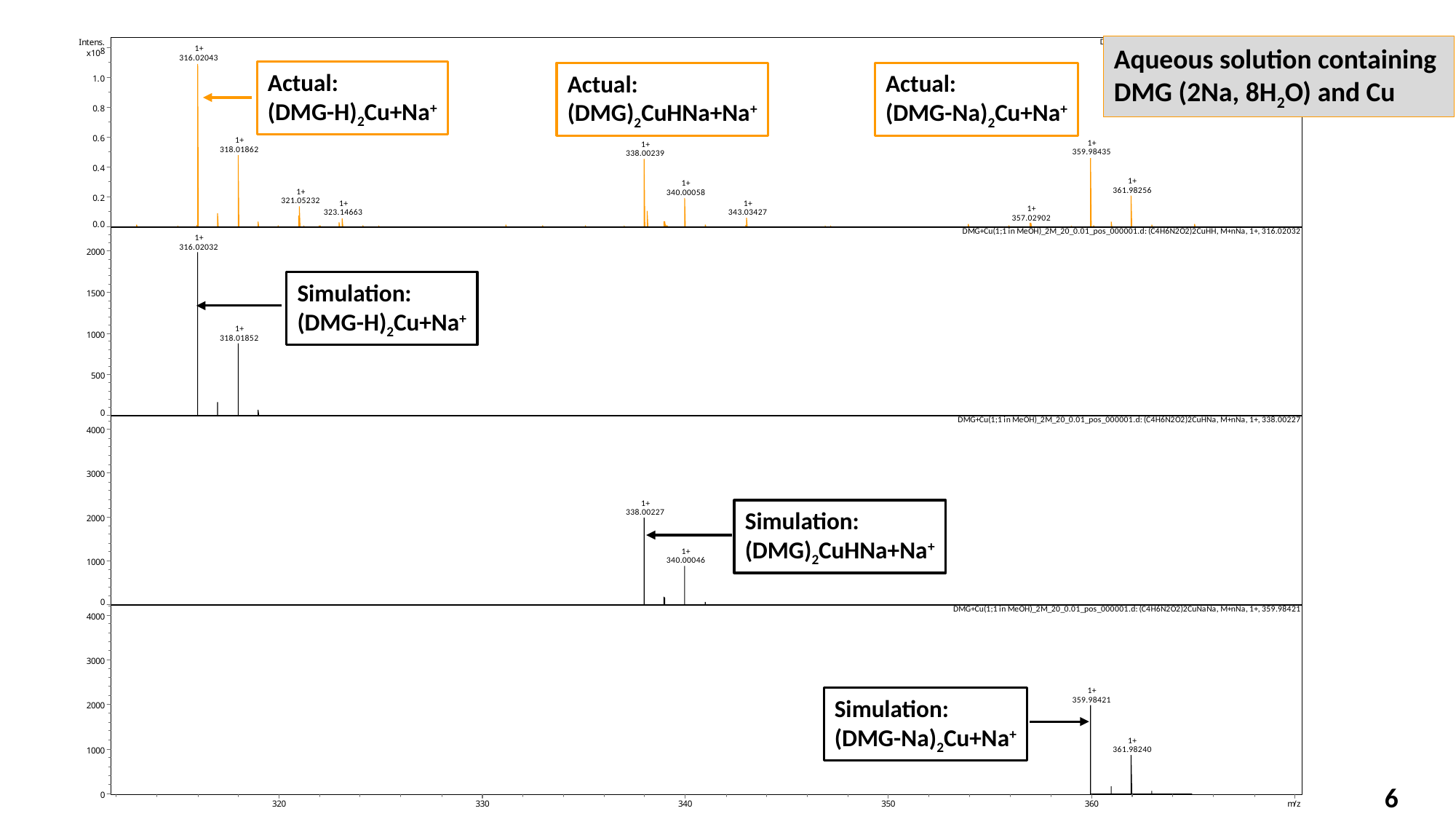

Aqueous solution containing
DMG (2Na, 8H2O) and Cu
Actual:
(DMG-H)2Cu+Na+
Actual:
(DMG-Na)2Cu+Na+
Actual:
(DMG)2CuHNa+Na+
Simulation:
(DMG-H)2Cu+Na+
Simulation:
(DMG)2CuHNa+Na+
Simulation:
(DMG-Na)2Cu+Na+
6

## Slide 8
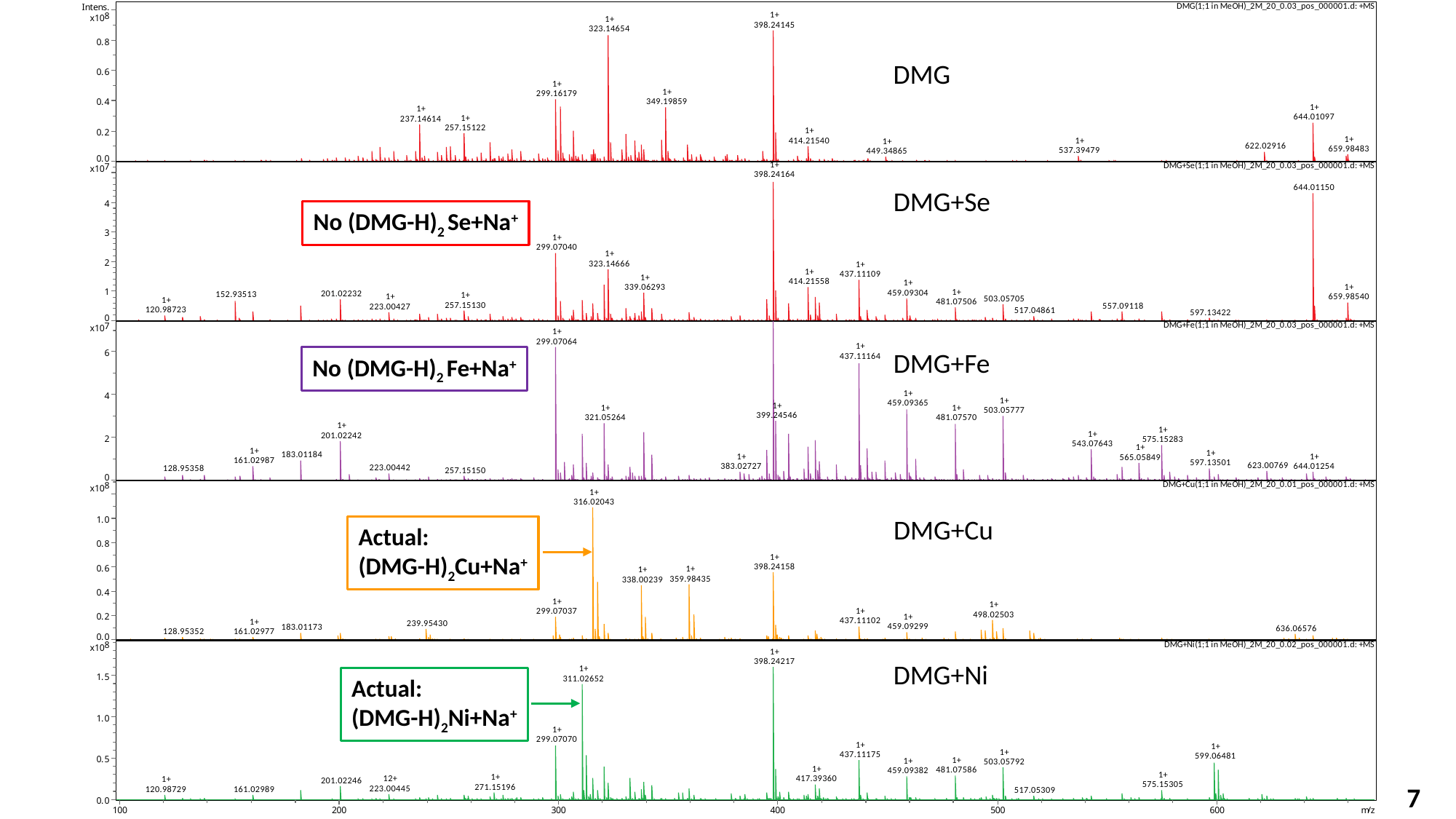

DMG
DMG+Se
No (DMG-H)2 Se+Na+
DMG+Fe
No (DMG-H)2 Fe+Na+
DMG+Cu
Actual:
(DMG-H)2Cu+Na+
DMG+Ni
Actual:
(DMG-H)2Ni+Na+
7
